# Supplementary material for: Resistance to Naïve and Formative Pluripotency Conversion in RSeT Human Embryonic Stem Cells
Source: bioRxiv. 2024 Apr 12:2024.02.16.580778. Originally published 2024 Feb 17. Preprint. [Version 2] doi: 10.1101/2024.02.16.580778 (PMC10896352; doi:10.1101/2024.02.16.580778)
Supplement: Supplement 1 [file media-1.pdf]

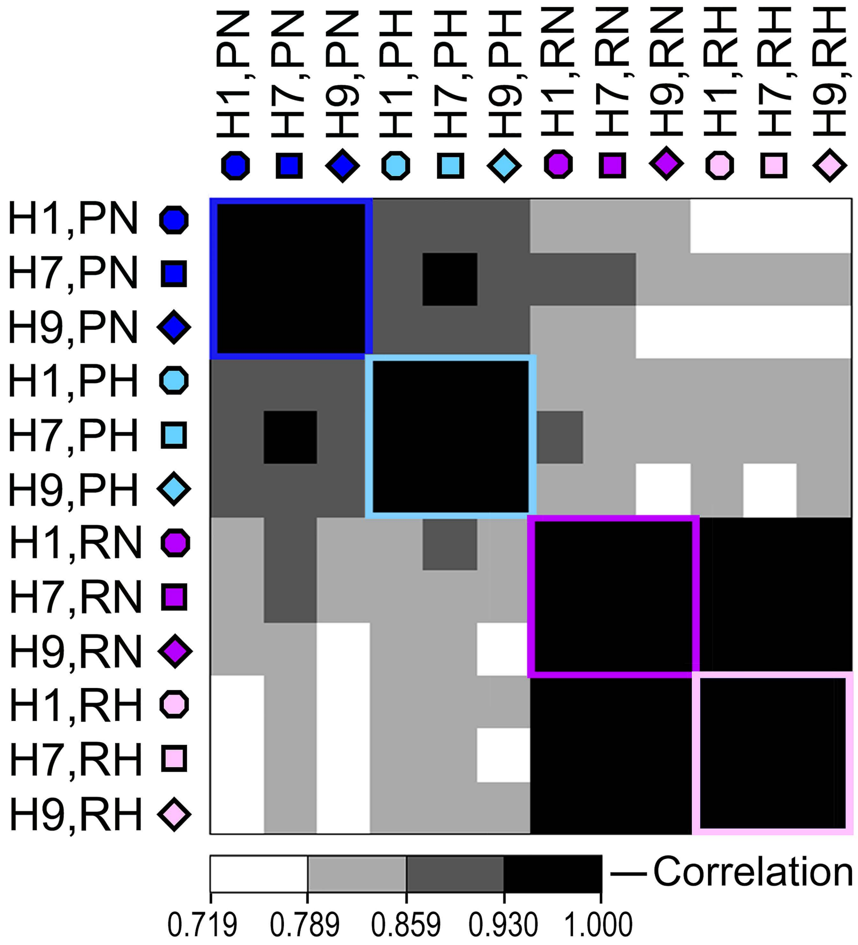

Figure S1. Pearson correlation heatmap depicts the 12 cell lines using log (base = 2) transformed and quantile normalized mRNA expression (related to Figure 2A).

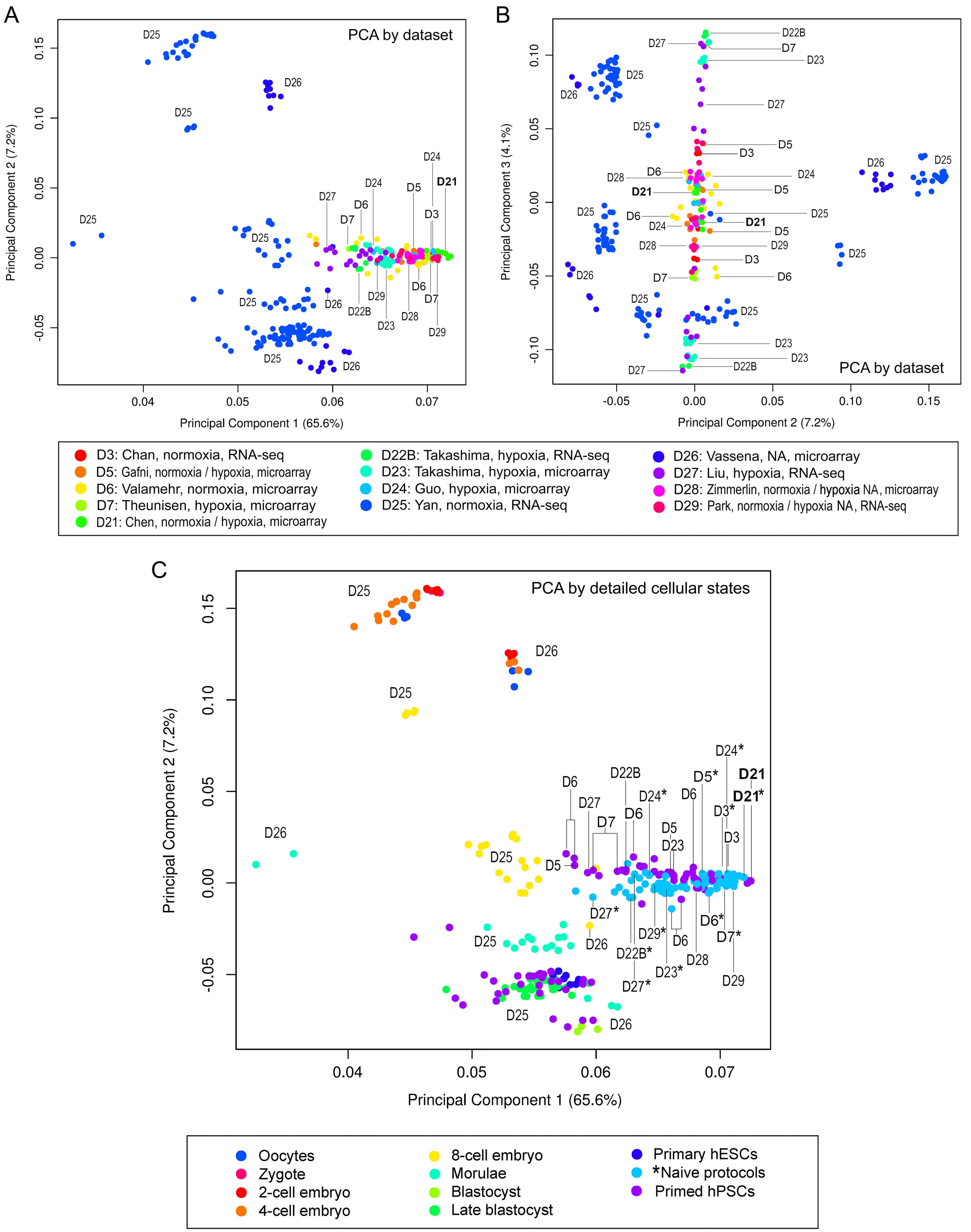

Figure S2. Principal component analysis (PCA) based on datasets and detailed cellular states (Related to Figure 3).

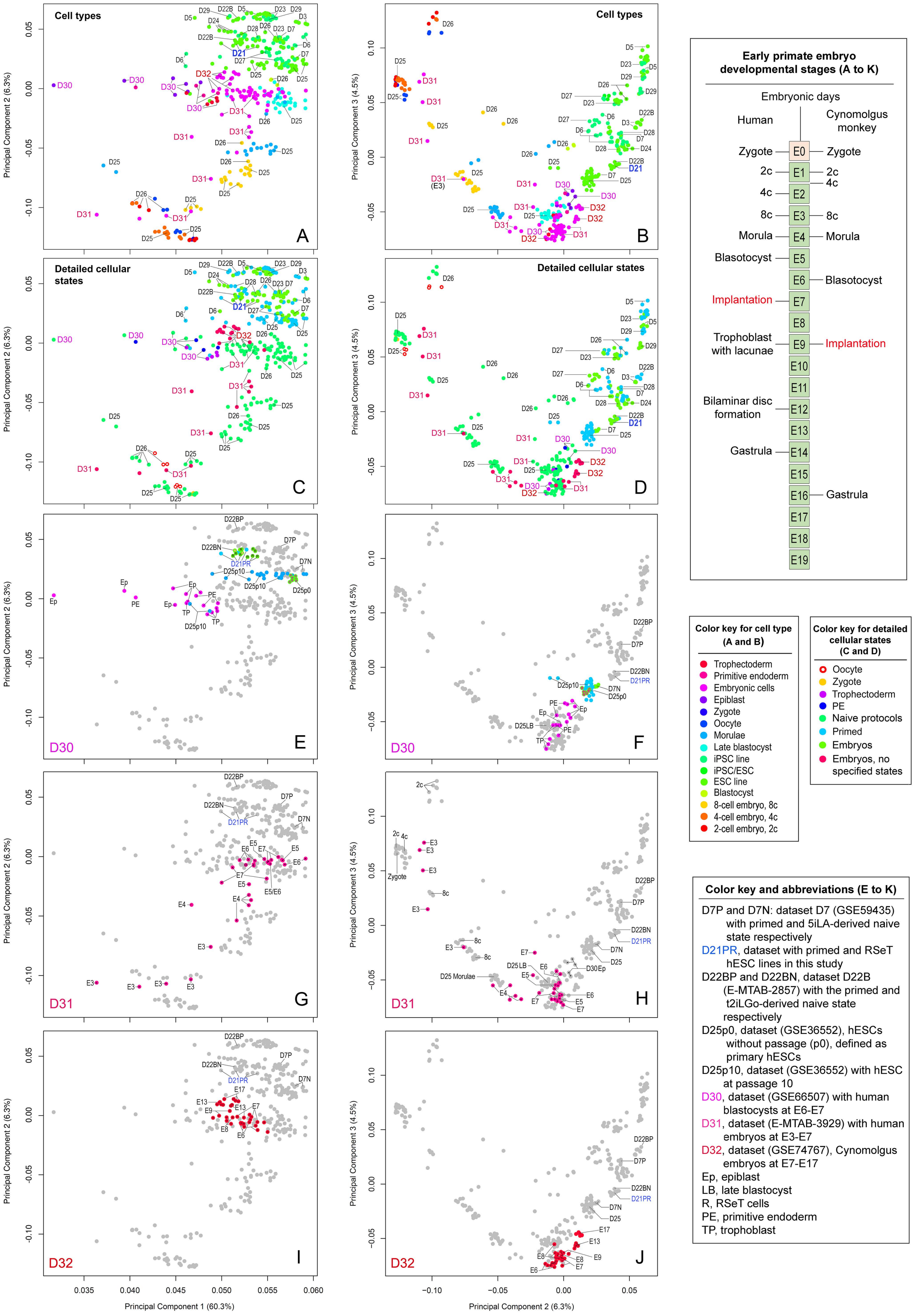

Figure S3. Principal component analysis (PCA) based on cell types, detailed cellular states, and early embryonic developmental stages (Related to Figure 3A and 3B).

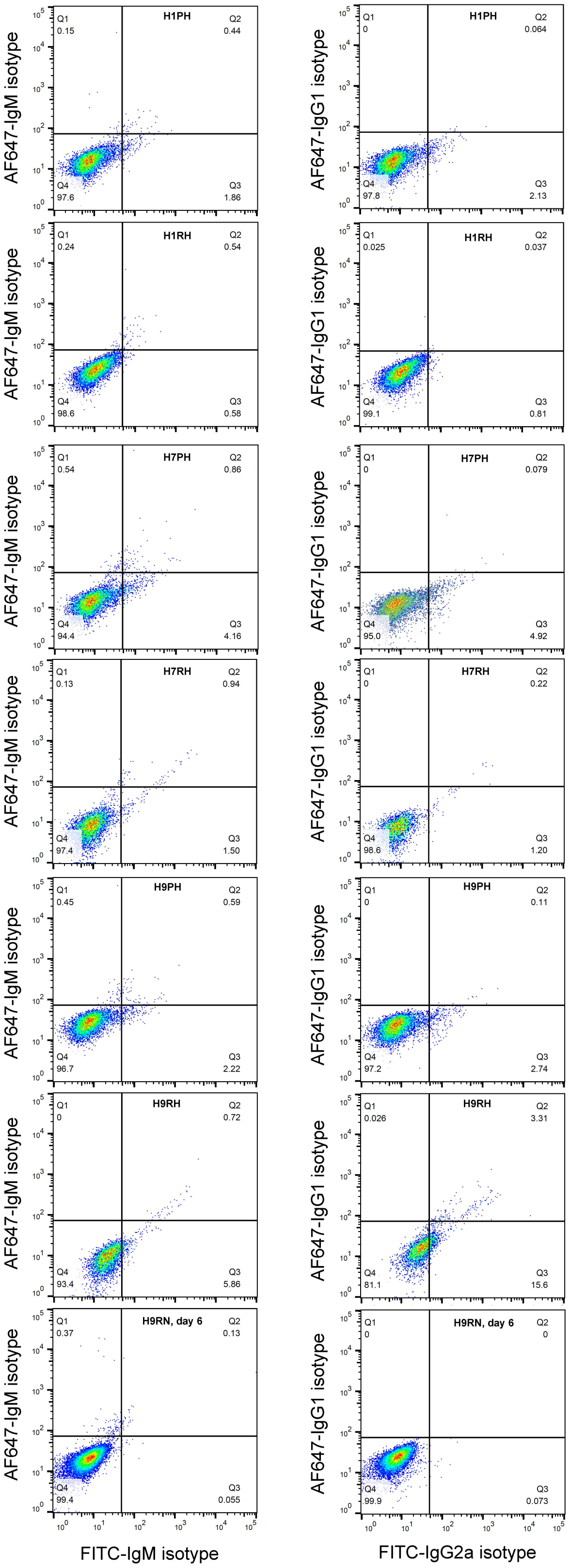

Figure S4. Representative flow cytometric controls depicting fluorescence labeled isotypoe antibodies used to gate positive antibody staining in primed and RSeT human embryonic stem cell lines (related to Figures 4 and 5)
