# Supplementary material for: Resistance to Naïve and Formative Pluripotency Conversion in RSeT Human Embryonic Stem Cells
Source: bioRxiv. 2024 Apr 12:2024.02.16.580778. Originally published 2024 Feb 17. Preprint. [Version 2] doi: 10.1101/2024.02.16.580778 (PMC10896352; doi:10.1101/2024.02.16.580778)
Supplement: Supplement 2 [file media-2.pdf]

Table S1. Gene expression signatures (n = 112) underlying various pluripotent states

Gene Symbols	Description (Based on GeneCards and Entrez of NCBI)	Gene expression probes	RSeT vs primed (n = 6, Fold changes)	RN vs PN (n = 3, fold changes)	RH vs PH (n = 3, fold changes)	Notes/ Comments with references[a]
**Inner cell mass (ICM) and other developmental markers not in implantation epiblast** (n = 5, *indicates *P* < 0.05)						
ATG2A	ICM expressed, not in the pre-implantation epiblast,	A_23_P361820	1.04	0.83	1.30	Pera & Rossant
ATG2B	ICM expressed, not in the pre-implantation epiblast,	A_23_P88163	1.70	2.06	1.41	Pera & Rossant
GATA3	GATA Binding Protein 3, ICM expressed, trophoblast marker	A_23_P75056	0.99	0.98	1.00	P list in Valamehr, Pera & Rossant
GATA6	A primitive endoderm marker	A_23_P304450	**0.19***	**0.08***	0.42	Pera & Rossant
MAGEA4	ICM expressed, not in the pre-implantation epiblast,	A_24_P185945	0.96	1.00	0.92	Pera & Rossant
**Naive-related or epiblast specific gene expression** (n = 60, *indicates *P* < 0.05)						
AHNAK	AHNAK Nucleoprotein	A_24_P943393	0.92	0.34	2.44	Chan, Epiblast specific,
ARRB1	Arrestin Beta 1	A_23_P203702	**0.20***	**0.11***	0.38	Chan, Epiblast specific
ATG13[b]	Autophagy Related 13	A_23_P95292	1.20	1.05	1.37	Tesar
CD44	CD44 Molecule (Indian Blood Group)	A_23_P24870	1.08	0.98	1.19	High in Gafni PSCs
CD9	CD9 Molecule	A_23_P76364	0.94	0.88	1.00	High in Takashima
CDH1	Cadherin 1	A_23_P206359	**1.58***	1.38	1.82	mESC specific
CLEC4D	C-Type Lectin Domain Family 4 Member D	A_23_P25235	1.09	1.14	1.04	Epiblast specific in Chan 3iL PSCs
COL1A1	Collagen Type I Alpha 1 Chain	A_23_P207520	**0.38***	0.28	0.50	High in Takashima & Chan PSCs
COMMD3	COMM Domain Containing 3	A_23_P138514	**0.63***	0.60	0.67	High in Takashima nPSCs
CTNNB1	Catenin Beta 1	A_23_P29495	0.83	0.82	0.84	High in Takashima nPSCs
DAZL	Deleted In Azoospermia Like	A_23_P212105	1.05	1.05	1.06	mESC specific
DNMT3L	DNA Methyltransferase 3 Like	A_23_P17673	**7.53***	5.42	10.47	Naive specific, Neri, Takashima, Theunissen
DPPA2	Developmental Pluripotency Associated 2	A_23_P405885	1.69	2.03	1.41	N list in Valamehr & Theunissen
DPPA5	Developmental Pluripotency Associated 5	A_32_P233950	**10.76***	11.85	9.78	List in Valamehr; Collier, Theunissen
DUSP6	Dual Specificity Phosphatase 6		**0.05***	**0.04***	**0.05***	
ESRRB	Estrogen Related Receptor Beta (not expressed in the human epiblast, High in t2iL+ dox inducible KLF2 and Nanog; Low in t2iL+ Gő)	A_24_P415928	1.01	1.03	1.00	List in Valamehr & Takashima; Pera & Rossant
ETV5	ETS Variant Transcription Factor 5	A_23_P9836	**0.58***	0.64	0.52	High in Gafni PSCs
FBXO15	F-Box Protein 15	A_23_P342709	**4.67***	**6.13***	**3.55***	mESC specific
FGF4	Fibroblast Growth Factor 4	A_24_P355720	**3.18***	2.55	3.95	List in Valamehr, Takashima, & Chan
FGF8	Fibroblast Growth Factor 8	A_23_P46829	0.69	0.70	0.69	High in Takashima & Chan PSCs
FN1	Fibronectin 1 **Transcriptionally regulated with naive protocols**	A_24_P119745	**0.36***	**0.19***	0.68	High in Tesar Takashima reports
GBX2	Gastrulation Brain Homeobox 2	A_23_P131183	0.97	0.94	1.00	mESC specific
GDF3	Growth Differentiation Factor 3	A_23_P72817	0.98	0.85	1.14	mESC specific, Chan
HORMAD1	HORMA Domain Containing 1	A_32_P199884	1.26	1.44	1.10	High in Tesar mESCs; Theunissen
ID3	Inhibitor of DNA Binding 3, HLH Protein	A_23_P137381	0.97	0.77	1.22	High in Gafni PSCs
ID4	Inhibitor Of DNA Binding 4, HLH Protein	A_23_P59375	1.05	1.08	1.03	List in Valamehr
IGFBP2	Insulin Like Growth Factor Binding Protein 2	A_23_P119943	0.97	1.25	0.75	High in Takashima nPSCs
IL6ST	Interleukin 6 Cytokine Family Signal Transducer,	A_32_P140656	0.93	0.73	1.19	Epiblast specific CD130 (IL6ST, LIF-coreceptor) expressed in human epiblast, Collier)
KIT	KIT Proto-Oncogene, Receptor Tyrosine Kinase	A_23_P110253	1.18	0.99	**1.42***	Takashima

KLF2	Kruppel Like Factor 2<br>Not expressed in human epiblast	A_23_P119196	**3.66***	2.51	**5.33***	In mESCs, Smith, Tesar, Takashima
KLF4	Kruppel Like Factor 4	A_23_P32233	**3.98***	**3.95***	**4.01***	Naive specific marker, Tesar & Theunissen
KLF5	Kruppel Like Factor 5	A_23_P53891	**3.50***	2.52	4.86	
LAMA1	Laminin Subunit Alpha 1	A_24_P100613	0.74	0.59	0.92	Takashima
LIFR	LIF Receptor Subunit Alpha	A_24_P397386	**0.51***	**0.35***	0.74	Takashima
LIMCH1	LIM and Calponin Homology Domains 1	A_32_P117354	**2.07***	1.95	2.20	Epiblast specific
MAEL	Maelstrom Spermatogenic Transposon Silencer	A_23_P114934	1.25	1.21	1.28	Theunissen
MFAP3L	Microfibril Associated Protein 3 Like	A_24_P76675	0.85	0.72	1.00	Epiblast specific
MICA	MHC Class I Polypeptide-Related Sequence A	A_23_P257516	**1.47***	1.40	**1.54***	
MICB	MHC Class I Polypeptide-Related Sequence B	A_23_P387471	**2.14***	2.08	2.20	
NANOG	Nanog Homeobox<br>**Transcriptionally regulated with naive protocols**	A_23_P204640	**0.64***	0.78	0.53	Collier & Theunissen
NR0B1	Nuclear Receptor Subfamily 0 Group B Member 1	A_23_P73632	**0.38***	**0.28***	0.51	Epiblast specific, Tesar
PIWIL2	Piwi Like RNA-Mediated Gene Silencing 2	A_32_P208654	0.58	0.86	0.39	Epiblast specific
POU5F1	**Transcriptionally regulated with naive protocols**	A_24_P214841	0.74	0.64	0.85	Theunissen
PRDM14	PR/SET Domain 14	A_23_P123488	1.00	1.18	0.85	Maintenance of pluripotency, Takashima
PYGL	Glycogen Phosphorylase L<br>**Transcriptionally regulated with naive protocols,** low in naïve mESCs	A_23_P48676	0.65	0.67	0.64	Chan, Tesar
REST	**Transcriptionally regulated with naive protocols**		**1.94***	1.90	1.99	Takashima
SLC25A16	Solute Carrier Family 25 Member 16	A_23_P423891	**3.04***	**3.11***	**2.98***	Epiblast specific
SMYD2	SET and MYND Domain Containing 2	A_23_P170587	1.38	1.07	1.77	Epiblast specific
SOAT1	Sterol O-Acyltransferase 1	A_32_P103131	**2.78***	2.11	3.65	Epiblast specific
SOX2	SRY-Box Transcription Factor 2	A_24_P379969	1.01	0.88	1.16	mESC specific, Takashima
STAT3	Signal Transducer and Activator of Transcription 3	A_23_P107206	**1.94***	1.55	**2.44***	List in Valamehr
TDGF1	Teratocarcinoma-Derived Growth Factor 1 (Known as Cripto-1)	A_23_P366376	0.77	0.86	0.68	Takashima
TERT	Telomerase Reverse Transcriptase	A_23_P110851	**0.59***	**0.69***	**0.51***	Takashima
TFCP2L1	Transcription Factor CP2 Like 1	A_23_P5301	**2.95***	3.66	2.37	List in Valamehr; Takashima, Theunissen
TFE3	Transcription Factor Binding to IGHM Enhancer 3	A_23_P84952	1.11	0.88	1.40	Takashima
UTF1	Undifferentiated Embryonic Cell Transcription Factor 1	A_23_P33141	1.43	1.50	1.36	Llist in Valamehr; mESC specific, Theunissen
XIST	X Inactive Specific Transcript	A_24_P500584	1.09	1.11	1.06	P list in Valamehr
ZFP42	ZFP42 Zinc Finger Protein, known as Rex1, a definitive naïve marker in the mouse	A_23_P395582	0.83	0.87	0.79	High in Takashima, Theunissen; Low in Gafni, Chan, & Ware PSCs
ZNF600	Zinc Finger Protein 600	A_23_P16006	1.22	1.01	1.48	Epiblast specific
**Formative (between pre-implantation and the E6.5 epiblast in mouse) gene expression** (n = 5, * indicates $P < 0.05$)						
DNMT3A	DNA Methyltransferase 3 Alpha<br>**Transcriptionally regulated with naïve protocols, decrease in naïve**	A_23_P154500	0.98	0.97	0.98	Smith review, Gafni
DNMT3B	DNA Methyltransferase 3 Beta<br>**Transcriptionally regulated with naïve protocols, decrease in naive**	A_23_P28953	0.99	0.96	1.02	Smith review; Decreased in Takashima, Gafni, & Neri
OTX2	Orthodenticle Homeobox 2<br>**Transcriptionally regulated with naive protocols**	A_23_P48663	**0.52***	0.52	0.52	Formative state, priming marker (Smith); Chan, Gafni, Theunissen, Valamehr, Ware
SALL2	Spalt Like Transcription Factor 2	A_23_P48585	**0.74***	**0.78***	0.59	Smith review
SOX3	SRY-Box Transcription Factor 3	A_23_P85218	0.60	0.82	0.44	Smith review
**Primed or metastable lineage specifier gene expression** (n = 37, * indicates $P < 0.05$)						
ACTC1	Actin Alpha Cardiac Muscle 1	A_23_P205894	**0.09***	**0.10***	**0.08***	

BMP2	Bone Morphogenetic Protein 2	A_23_P143331	**0.28***	0.20	0.40	
CD47	CD47 Molecule	A_23_P6935	0.77	0.82	0.72	
CDH11	Cadherin 11	A_23_P152305	1.33	1.54	1.15	P list in Valamehr
CER1	Cerberus 1, DAN Family BMP Antagonist	A_23_P329798	**0.27***	0.34	**0.22***	High in EpiSCs (Tesar & Brons); low in Chan & Takashima PSCs
COL13A1	Collagen Type XIII Alpha 1 Chain	A_23_P1331	**0.28***	0.27	**0.28***	P list in Valamehr
CXCR4	C-X-C Motif Chemokine Receptor 4	A_23_P102000	**0.19***	**0.15***	0.25	P list in Valamehr
CYP2B6	Cytochrome P450 Family 2 Subfamily B Member 6	A_24_P339514	**0.32***	0.40	0.25	P list in Valamehr
DLL1	Delta Like Canonical Notch Ligand 1	A_23_P167920	**2.86***	2.29	3.57	
DNMT1	DNA Methyltransferase 1	A_24_P408083	0.90	0.80	1.01	Zhao
EGR1	Early Growth Response 1	A_23_P214080	**0.01***	**0.01***	**0.01***	P list in Valamehr
EOMES	Eomesodermin	A_24_P97374	**0.18***	0.09	0.36	
ERBB4	Erb-B2 Receptor Tyrosine Kinase 4	A_32_P183765	**0.08***	**0.07***	**0.09***	P list in Valamehr
FLT1	Fms Related Receptor Tyrosine Kinase 1	A_24_P576191	**0.22***	0.34	0.15	
FOXA2	Forkhead Box A2	A_24_P365515	**0.15***	**0.07***	0.36	
GAL	Galanin And GMAP Prepropeptide	A_23_P374844	**0.22***	**0.18***	0.27	P list in Valamehr
GSC	Goosecoid Homeobox	A_23_P76774	**0.07***	**0.06***	**0.08***	P list in Valamehr
HEPH	Hephaestin	A_24_P399980	**0.74***	0.73	0.74	P list in Valamehr
HES1	Hes Family BHLH Transcription Factor 1	A_23_P17998	**0.26***	0.31	0.22	P list in Valamehr
HHEX	Hematopoietically Expressed Homeobox	A_23_P47034	0.70	0.50	0.97	Pera & Rossant
LEFTY1	Left-Right Determination Factor 1	A_23_P160336	**0.04***	0.08	0.02	P list in Valamehr Mallon, Chen
LEFTY2	Left-Right Determination Factor 2	A_23_P137573	**0.18***	0.27	0.13	Mallon, Chen
MEGF10	Multiple EGF Like Domains 10	A_24_P7192	1.74	2.15	1.41	P list in Valamehr
MEIS1	Meis Homeobox 1 **Transcriptionally regulated with naive protocols**	A_24_P319736	1.24	1.55	1.00	Low in Gafni PSCs
MEIS2	Meis Homeobox 2 **Transcriptionally regulated with naive protocols**	A_24_P82200	1.59	1.62	1.56	Low in Gafni PSCs
NIPBL	NIPBL Cohesin Loading Factor	A_24_P180383	0.83	0.81	0.85	P list in Valamehr
**NODAL**	**Transcriptionally regulated with naive protocols**	A_23_P127322	0.48	0.59	0.38	Pera & Rossant
NR2F2	Nuclear Receptor Subfamily 2 Group F Member 2	A_23_P88589	1.33	1.76	1.00	P list in Valamehr
PCDH10	Protocadherin 10	A_32_P168605	0.79	0.63	1.00	P list in Valamehr
PITX2	Paired Like Homeodomain 2	A_23_P167367	**0.06***	0.08	**0.05***	P list in Valamehr
RORA	RAR Related Orphan Receptor A	A_23_P26124	0.58	0.70	0.48	P list in Valamehr
SOX1	SRY-Box Transcription Factor 1	A_24_P112803	1.37	1.37	1.37	Pera & Rossant
SOX11	SRY-Box Transcription Factor 11 associated with neuron differentiation	A_24_P302584	1.30	1.40	1.21	Low in Gafni PSCs
TGFB2	Transforming Growth Factor Beta 2	A_24_P148261	**0.69***	0.82	0.58	
TOX	Thymocyte Selection Associated High Mobility Group Box	A_24_P226755	1.21	1.33	1.10	P list in Valamehr
ZIC1	Zic Family Member 1	A_23_P367618	1.00	1.00	1.00	Low in Gafni PSCs
ZIC2	Zic Family Member 2	A_23_P36972	0.83	0.89	0.77	Priming marker (Smith)
**Other X-lined or imprinted gene expression** (n = 5, * indicates *P* < 0.05)						
DLK1	PEG9, Paternally Expressed 10, Known as DLK1, Delta Like Non-Canonical Notch Ligand 1	A_24_P236251	3.41	2.56	4.54	Low in naive & high in primed states in Gafni
FMR1	FMRP Translational Regulator 1, X-linked	A_24_P93967	1.30	1.80	0.93	
H19	H19, Imprinted Maternally Expressed	A_24_P52697	1.60	0.90	2.87	
MEG3	MEG3, Paternally Imprinted gene, Known as GTL2 **Transcriptionally regulated with naive protocols**	A_24_P272993	1.52	1.25	1.85	High in naïve (Collier)
MEST	MEST, Mesoderm specific, imprinted, preferentially paternally expressed	A_23_P156970	0.92	0.92	0.93	

FOOTNOTES

[a] Based on the references as detailed below.

[b] Transcriptionally regulated genes are indicated in bold gene symbols, with up-regrated gene in red and down-regulated gene in blue colors.

* Indicates *P* values < 0.05 with two-tailed Student *t*-test, for fold changes of gene expression in microarray.

Additional abbreviations: EPS, epiblast specific gene signature; mESCs, naive mouse embryonic stem cells; N, naive-related genes; nPSCs, naive pluripotent stem cells; PSCs, pluripotent stem cells; P, primed or primed genes or cells.

REFERENCES

Neri, F. et al. Genome-wide analysis identifies a functional association of Tet1 and Polycomb repressive complex 2 in mouse embryonic stem cells. Genome Biol 14, R91 (2013). https://doi.org:10.1186/gb-2013-14-8-r91

Brons, I. G. et al. Derivation of pluripotent epiblast stem cells from mammalian embryos. Nature 448, 191-195 (2007). https://doi.org:10.1038/nature05950

Chan, Y. S. et al. Induction of a human pluripotent state with distinct regulatory circuitry that resembles preimplantation epiblast. Cell Stem Cell 13, 663-675 (2013). https://doi.org:10.1016/j.stem.2013.11.015

Collier, A. J. et al. Comprehensive Cell Surface Protein Profiling Identifies Specific Markers of Human Naive and Primed Pluripotent States. Cell Stem Cell 20, 874-890 e877 (2017). https://doi.org:10.1016/j.stem.2017.02.014

Gafni, O. et al. Derivation of novel human ground state naive pluripotent stem cells. Nature 504, 282-286 (2013). https://doi.org:10.1038/nature12745

Liao, J. et al. Targeted disruption of DNMT1, DNMT3A and DNMT3B in human embryonic stem cells. Nat Genet 47, 469-478 (2015). https://doi.org:10.1038/ng.3258

Chen, K. G. et al. Non-colony type monolayer culture of human embryonic stem cells. Stem Cell Res 9, 237-248 (2012). https://doi.org:10.1016/j.scr.2012.06.003

Mallon, B. S. et al. StemCellDB: the human pluripotent stem cell database at the National Institutes of Health. Stem Cell Res 10, 57-66 (2013). https://doi.org:10.1016/j.scr.2012.09.002

Pera, M. F. & Rossant, J. The exploration of pluripotency space: Charting cell state transitions in peri-implantation development. Cell Stem Cell 28, 1896-1906 (2021). https://doi.org:10.1016/j.stem.2021.10.001

Smith, A. Formative pluripotency: the executive phase in a developmental continuum. Development 144, 365-373 (2017). https://doi.org:10.1242/dev.142679

Takashima, Y. et al. Resetting transcription factor control circuitry toward ground-state pluripotency in human. Cell 158, 1254-1269 (2014). https://doi.org:10.1016/j.cell.2014.08.029

Tesar, P. J. et al. New cell lines from mouse epiblast share defining features with human embryonic stem cells. Nature 448, 196-199 (2007). https://doi.org:10.1038/nature05972

Theunissen, T. W. et al. Molecular Criteria for Defining the Naive Human Pluripotent State. Cell Stem Cell 19, 502-515 (2016). https://doi.org:10.1016/j.stem.2016.06.011

Theunissen, T. W. et al. Systematic identification of culture conditions for induction and maintenance of naive human pluripotency. Cell Stem Cell 15, 471-487 (2014). https://doi.org:10.1016/j.stem.2014.07.002

Valamehr, B. et al. Platform for induction and maintenance of transgene-free hiPSCs resembling ground state pluripotent stem cells. Stem Cell Reports 2, 366-381 (2014). https://doi.org:10.1016/j.stemcr.2014.01.014

Weinberger, L., Ayyash, M., Novershtern, N. & Hanna, J. H. Dynamic stem cell states: naive to primed pluripotency in rodents and humans. Nat Rev Mol Cell Biol 17, 155-169 (2016). https://doi.org:10.1038/nrm.2015.28
