# Supplementary material for: Resistance to Naïve and Formative Pluripotency Conversion in RSeT Human Embryonic Stem Cells
Source: bioRxiv. 2024 Apr 12:2024.02.16.580778. Originally published 2024 Feb 17. Preprint. [Version 2] doi: 10.1101/2024.02.16.580778 (PMC10896352; doi:10.1101/2024.02.16.580778)
Supplement: Supplement 3 [file media-3.pdf]

KEY RESOURCES TABLE

REAGENT or RESOURCE	SOURCE	IDENTIFIER
**Chemicals**		
Dimethyl sulfoxide (DMSO)	Sigma	D2650
Dorsomorphin	Sigma	P5499
Erlotinib HCl (OSI-744)	Selleck Chemicals	S1023
Gö6983	Sigma	G1918-1MG
Gö6983	Sigma	G1918
JAK inhibitor I	EMD4 Biosciences	420099
Paraformaldehyde (PFA)	Electron Microscopy Sciences	15710
PD173074	Selleck Chemicals	S1264
SB431542	Tocris Bioscience	1614
Y-27632	EMD4 Biosciences	688000
Y-27632 dihydrochloride	Tocris Bioscience	1254
β-mercaptoethanol (14.3 M)	Sigma	M6250
**Antibodies**		
BD Pharmingen™ Alexa Fluor® 647 Mouse Anti-Human CD130; Mouse BALB/c IgG1, κ; Clone: AM64	BD Biosciences	564151
BD Pharmingen™ Alexa Fluor® 647 Mouse Anti-Human CD75/CD75s, IgM, κ, Clone: ZB55	BD Biosciences	566350
BD Pharmingen™ Alexa Fluor® 647 Mouse Anti-Human SUSD2, IgG1, κ, Clone: W5C5	BD Biosciences	566657
BD Pharmingen™ Alexa Fluor® 647 Mouse IgM, κ Isotype Control, Clone: G155-228	BD Biosciences	560806
BD Pharmingen™ FITC Mouse Anti-Human CD57 Mouse IgM, κ, Clone: NK-1	BD Biosciences	555619
BD Pharmingen™ FITC Mouse IgG2a, κ Isotype Control, Clone: G155-178	BD Biosciences	555573
BD Pharmingen™ FITC Mouse IgM, κ Isotype Control, Clone: G155-228	BD Biosciences	551448
BD Phosflow™ Alexa Fluor® 647 Mouse IgG1 κ Isotype control, MOPC-21	BD Biosciences	557783
BD™ CD24 FITC, Isotype: Mouse IgG2a, κ, Clone: ML5	BD Biosciences	655154
Goat anti-Mouse IgG1 Secondary Antibody, Alexa Fluor® 488 conjugate	Thermo Fisher Scientific	A-21121

Goat anti-Mouse IgG1 Secondary Antibody, Alexa Fluor® 647 conjugate	Thermo Fisher Scientific	A-21240
Goat anti-Mouse IgG2a Secondary Antibody, Alexa Fluor® 555 conjugate	Thermo Fisher Scientific	A-21137
Goat anti-Mouse IgG2a Secondary Antibody, Alexa Fluor® 647 conjugate	Thermo Fisher Scientific	A-21241
Goat anti-Mouse IgG2b Secondary Antibody, Alexa Fluor® 647 conjugate	Thermo Fisher Scientific	A-21242
Goat anti-Mouse IgM Heavy Chain Secondary Antibody, Alexa Fluor® 647 conjugate	Thermo Fisher Scientific	A-21238
Goat anti-Mouse IgM Heavy Chain Secondary Antibody, Alexa Fluor® 647 conjugate	Thermo Fisher Scientific	A-21238
NANOG (rabbit IgG)	ReproCELL Inc, Japan	RCAB0004P-F
Oct-4, mouse IgG2b	Santa Cruz Biotechnology	sc-5279
Pharmingen™ FITC Mouse Anti-Human CD90, Isotype: Mouse IgM, κ; Clone: 5E10	BD Biosciences	561969
SSEA-1, mouse IgM	Santa Cruz Biotechnology	sc-21702
SSEA-4, mouse IgG3	Santa Cruz Biotechnology	sc-21704
Tra-1-60, mouse IgM	Santa Cruz Biotechnology	sc-21705
Tra-1-81, mouse IgM	Santa Cruz Biotechnology	sc-21706
**Cell Culture Reagents**		
Accutase™	Innovative Cell Technologies	AT-104
BD Falcon Cell Strainer	BD Bioscience	352340
Bovine Albumin Fraction V (7.5% solution)	Thermo Fisher Scientific	15260037
CryoStor CS10	StemCell Technologies	7930
DMEM/F12, no HEPES	Thermo Fisher Scientific	11320-082
DMEM/F12, with HEPES	Thermo Fisher Scientific	11330–032
Dulbecco's Phosphate-Buffered Saline	Thermo Fisher Scientific	14190-144
Faxitron Cabinet X-ray System	Faxitron X-ray Corporation, Wheeling, IL	Model RX-650
Fetal Bovine Serum (FBS), certified, heat inactivated	Thermo Fisher Scientific	10082147
Heat-inactivated fetal bovine serum (FBS)	Hyclone (Logan Utah)	SH30071-03
hESC-qualified Matrigel	BD Bioscience	354277
Knockout Serum Replacer	Thermo Fisher Scientific	10828–028
L-Glutamine (200 mM)	Thermo Fisher Scientific	25030–081
MEM non-essential amino acids solution (100X)	Thermo Fisher Scientific	11140050
mTeSR1 and Supplements	StemCell Technologies	5850
MULTIWELL six-well plates	Becton Dickinson Labware	353046

Nalgene 5100-0001 Cryo 1°C	Thermo Fisher Scientific	C6516F-1
RSeT™ Medium (2-Component)	StemCell Technologies	05978
Thermo Scientific Nunc Thermanox Coverslips (25 mm diameter; 500/cs)	Thermo Fisher Scientific	174985 (25 mm diameter)
TrypLE™ Express	Thermo Fisher Scientific	REF 12604-013
Trypsin	Thermo Fisher Scientific	25300-054
**Critical Commercial Assays**		
Countess™ automated cell counter (assay)	Thermo Fisher Scientific	C10227
**Deposited Data**		
Microarray	Agilent Technologies, Inc.	In this study
**Experimental Models: Cell Lines**		
H1 (WA01)	WiCell Inc.	NIHhESC-10-0043
H7 (WA07)	WiCell Inc.	NIHhESC-10-0061
H9 (WA09)	WiCell Inc.	NIHhESC-10-0062
**Real-time PCR Reagents**		
Nuclease-free water	Thermo Fisher Scientific	R0582
SuperScript® VILO™ cDNA Synthesis Kit	Thermo Fisher Scientific	11754050
TagMan Assay ID Hs99999903_m1 for *ACTB*	Thermo Fisher Scientific	4331182
TagMan Assay ID Hs99999905_m1 for *GAPDH*	Thermo Fisher Scientific	4453320
TaqMan® Fast Advanced Master Mix	Thermo Fisher Scientific	4444557
TE, pH 8.0, RNase-free	Thermo Fisher Scientific	AM9849
**Recombinant DNA**		
None		
**Software and Algorithms**		
FlowJo	BD Biosciences	Version 10.9.0
ImageJ	NIH, Bethesda, USA	
QuantStudio™ 6 and 7 Flex Real-Time PCR System Software	Thermo Fisher Scientific	Publication Number 4489822
R software package	http://cran.r-project.org/	
SC3 consensus clustering	Bioconductor (http://bioconductor.org)	Version 3.12
