# Supplementary material for: Resistance to Naïve and Formative Pluripotency Conversion in RSeT Human Embryonic Stem Cells
Source: bioRxiv. 2024 Feb 17:2024.02.16.580778. Preprint. [Version 1] doi: 10.1101/2024.02.16.580778 (PMC10896352; doi:10.1101/2024.02.16.580778)
Supplement: Supplementary file 1 [file media-5.docx]

**Table S1.** **Gene expression signatures underlying various pluripotent states**

| **Gene Symbols** | **Description** **(Based on GeneCards and** **Entrez of NCBI)** | **Gene expression** **probes** | **RSeT** **vs primed** **(n = 6, Fold changes)** | **RN vs PN** **(n = 3, fold changes)** | **RH vs PH** **(n = 3,** **fold changes)** | **Notes/** **Comments with references**^**a**^ |
| --- | --- | --- | --- | --- | --- | --- |
| **Inner cell mass (ICM) and other developmental markers not in implantation epiblast** (n = 5, *indicates *P* < 0.05) |  |  |  |  |  |  |
| *ATG2A* | ICM expressed, not in the pre-implantation epiblast, | A_23_P361820 | 1.04 | 0.83 | 1.30 | Pera & Rossant |
| *ATG2B* | ICM expressed, not in the pre-implantation epiblast, | A_23_P88163 | 1.70 | 2.06 | 1.41 | Pera & Rossant |
| *GATA3* | GATA Binding Protein 3, ICM expressed, trophoblast marker | A_23_P75056 | 0.99 | 0.98 | 1.00 | P list in Valamehr, Pera & Rossant |
| *GATA6* | A primitive endoderm marker | A_23_P304450 | **0.19*** | **0.08*** | 0.42 | Pera & Rossant |
| *MAGEA4* | ICM expressed, not in the pre-implantation epiblast, | A_24_P185945 | 0.96 | 1.00 | 0.92 | Pera & Rossant |
| **Naive-related or epiblast specific gene expression** (n = 58, *indicates *P* < 0.05) |  |  |  |  |  |  |
| *AHNAK* | AHNAK Nucleoprotein | A_24_P943393 | 0.92 | 0.34 | 2.44 | Chan, Epiblast specific, |
| *ARRB1* | Arrestin Beta 1 | A_23_P203702 | **0.20*** | **0.11*** | 0.38 | Chan, Epiblast specific |
| ***ATG13***^***b***^ | Autophagy Related 13 | A_23_P95292 | 1.20 | 1.05 | 1.37 | Tesar |
| ***CD44*** | CD44 Molecule (Indian Blood Group) | A_23_P24870 | 1.08 | 0.98 | 1.19 | High in Gafni PSCs |
| ***CD9*** | CD9 Molecule | A_23_P76364 | 0.94 | 0.88 | 1.00 | High in Takashima |
| ***CDH1*** | Cadherin 1 | A_23_P206359 | **1.58*** | 1.38 | 1.82 | mESC specific |
| ***CLEC4D*** | C-Type Lectin Domain Family 4 Member D | A_23_P25235 | 1.09 | 1.14 | 1.04 | Epiblast specific in Chan 3iL PSCs |
| ***COL1A1*** | Collagen Type I Alpha 1 Chain | A_23_P207520 | **0.38*** | 0.28 | 0.50 | High in Takashima & Chan PSCs |
| ***COMMD3*** | COMM Domain Containing 3 | A_23_P138514 | **0.63*** | 0.60 | 0.67 | High in Takashima nPSCs |
| ***CTNNB1*** | Catenin Beta 1 | A_23_P29495 | 0.83 | 0.82 | 0.84 | High in Takashima nPSCs |
| ***DAZL*** | Deleted In Azoospermia Like | A_23_P212105 | 1.05 | 1.05 | 1.06 | mESC specific |
| ***DNMT3L*** | DNA Methyltransferase 3 Like | A_23_P17673 | **7.53*** | 5.42 | 10.47 | Naive specific, Neri, Takashima, Theunissen |
| ***DPPA2*** | Developmental Pluripotency Associated 2 | A_23_P405885 | 1.69 | 2.03 | 1.41 | N list in Valamehr & Theunissen |
| ***DPPA5*** | Developmental Pluripotency Associated 5 | A_32_P233950 | **10.76*** | 11.85 | 9.78 | List in Valamehr; Collier, Theunissen |
| ***DUSP6*** | Dual Specificity Phosphatase 6 |  | **0.05*** | **0.04*** | **0.05*** |  |
| ***ESRRB*** | Estrogen Related Receptor Beta # (ICM expressed, not in the human epiblast, # High in t2iL+ dox inducible KLF2 and Nanog; Low in t2iL+ Gὄ) |  |  |  |  | List in Valamehr & Takashima; Pera & Rossant |
| ***ETV5*** | ETS Variant Transcription Factor 5 | A_23_P9836 | **0.58*** | 0.64 | 0.52 | High in Gafni PSCs |
| *FBXO15* | F-Box Protein 15 | A_23_P342709 | **4.67*** | **6.13*** | **3.55*** | mESC specific |
| ***FGF4*** | Fibroblast Growth Factor 4 | A_24_P355720 | **3.18*** | 2.55 | 3.95 | List in Valamehr, Takashima, & Chan |
| ***FGF8*** | Fibroblast Growth Factor 8 | A_23_P46829 | 0.69 | 0.70 | 0.69 | High in Takashima & Chan PSCs |
| ***FN1*** | Fibronectin 1 **Transcriptionally regulated with naive protocols** | A_24_P119745 | **0.36*** | **0.19*** | 0.68 | High in Tesar Takashima reports |
| ***GBX2*** | Gastrulation Brain Homeobox 2 | A_23_P131183 | 0.97 | 0.94 | 1.00 | mESC specific |
| ***GDF3*** | Growth Differentiation Factor 3 | A_23_P72817 | 0.98 | 0.85 | 1.14 | mESC specific, Chan |
| ***HORMAD1*** | HORMA Domain Containing 1 | A_32_P199884 | 1.26 | 1.44 | 1.10 | High in Tesar mESCs; Theunissen |
| ***ID3*** | Inhibitor of DNA Binding 3, HLH Protein | A_23_P137381 | 0.97 | 0.77 | 1.22 | High in Gafni PSCs |
| *ID4* | Inhibitor Of DNA Binding 4, HLH Protein | A_23_P59375 | 1.05 | 1.08 | 1.03 | List in Valamehr |
| ***IGFBP2*** | Insulin Like Growth Factor Binding Protein 2 | A_23_P119943 | 0.97 | 1.25 | 0.75 | High in Takashima nPSCs |
| ***IL6ST*** | Interleukin 6 Cytokine Family Signal Transducer, | A_32_P140656 | 0.93 | 0.73 | 1.19 | Epiblast specific CD130 (IL6ST, LIF-coreceptor) expressed in human epiblast, Collier) |
| ***KIT*** | KIT Proto-Oncogene, Receptor Tyrosine Kinase | A_23_P110253 | 1.18 | 0.99 | **1.42*** | Takashima |
| ***KLF2*** | Kruppel Like Factor 2 Not expressed in human epiblast | A_23_P119196 | **3.66*** | 2.51 | **5.33*** | In mESCs, Smith, Tesar, Takashima |
| ***KLF4*** | Kruppel Like Factor 4 | A_23_P32233 | **3.98*** | **3.95*** | **4.01*** | Naive specific marker, Tesar & Theunissen |
| ***KLF5*** | Kruppel Like Factor 5 | A_23_P53891 | **3.50*** | 2.52 | 4.86 |  |
| ***KLF17*** | Human naïve transcriptomic marker, reviewed by Zhou J et al. Cell Reports, 2023. |  |  |  |  |  |
| ***LAMA1*** | Laminin Subunit Alpha 1 | A_24_P100613 | 0.74 | 0.59 | 0.92 | Takashima |
| ***LIFR*** | LIF Receptor Subunit Alpha | A_24_P397386 | **0.51*** | **0.35*** | 0.74 | Takashima |
| ***LIMCH1*** | LIM and Calponin Homology Domains 1 | A_32_P117354 | **2.07*** | 1.95 | 2.20 | Epiblast specific |
| ***MAEL*** | Maelstrom Spermatogenic Transposon Silencer | A_23_P114934 | 1.25 | 1.21 | 1.28 | Theunissen |
| *MFAP3L* | Microfibril Associated Protein 3 Like | A_24_P76675 | 0.85 | 0.72 | 1.00 | Epiblast specific |
| *MICA* | MHC Class I Polypeptide-Related Sequence A | A_23_P257516 | **1.47*** | 1.40 | **1.54*** |  |
| *MICB* | MHC Class I Polypeptide-Related Sequence B | A_23_P387471 | **2.14*** | 2.08 | 2.20 |  |
| ***NANOG*** | Nanog Homeobox **Transcriptionally regulated with naive protocols** | A_23_P204640 | **0.64*** | 0.78 | 0.53 | Collier & Theunissen |
| ***NLRP7*** | Human naïve transcriptomic marker, reviewed by Zhou J et al. Cell Reports, 2023. Huang K et al CSC 2014; Yan L et al Nat Struct Mol Biol 2013. |  |  |  |  |  |
| ***NR0B1*** | Nuclear Receptor Subfamily 0 Group B Member 1 | A_23_P73632 | **0.38*** | **0.28*** | 0.51 | Epiblast specific, Tesar |
| *PIWIL2* | Piwi Like RNA-Mediated Gene Silencing 2 | A_32_P208654 | 0.58 | 0.86 | 0.39 | Epiblast specific |
| ***POU5F1*** | **Transcriptionally regulated with naive protocols** | A_24_P214841 | 0.74 | 0.64 | 0.85 | Theunissen |
| *PRDM14* | PR/SET Domain 14 | A_23_P123488 | 1.00 | 1.18 | 0.85 | Maintenance of pluripotency, Takashima |
| ***PYGL*** | Glycogen Phosphorylase L **Transcriptionally regulated with naive protocols,** low in naïve mESCs | A_23_P48676 | 0.65 | 0.67 | 0.64 | Chan, Tesar |
| ***REST*** | **Transcriptionally regulated with naive protocols** |  | **1.94*** | 1.90 | 1.99 | Takashima |
| *SLC25A16* | Solute Carrier Family 25 Member 16 | A_23_P423891 | **3.04*** | **3.11*** | **2.98*** | Epiblast specific |
| *SMYD2* | SET and MYND Domain Containing 2 | A_23_P170587 | 1.38 | 1.07 | 1.77 | Epiblast specific |
| *SOAT1* | Sterol O-Acyltransferase 1 | A_32_P103131 | **2.78*** | 2.11 | 3.65 | Epiblast specific |
| ***SOX2*** | SRY-Box Transcription Factor 2 | A_24_P379969 | 1.01 | 0.88 | 1.16 | mESC specific, Takashima |
| *STAT3* | Signal Transducer and Activator of Transcription 3 | A_23_P107206 | **1.94*** | 1.55 | **2.44*** | List in Valamehr |
| ***TDGF1*** | Teratocarcinoma-Derived Growth Factor 1 (Known as Cripto-1) | A_23_P366376 | 0.77 | 0.86 | 0.68 | Takashima |
| ***TERT*** | Telomerase Reverse Transcriptase | A_23_P110851 | **0.59*** | **0.69*** | **0.51*** | Takashima |
| ***TFCP2L1*** | **Transcription Factor CP2 Like 1;** # Involved in regu of transcription by RNA Pol II # Predicted located at cyto, memb, chromatin, nucleus. # With KLF2 acts major effector of downstream of LIF/STAT3 & Wnt/b_catenin # Requirement for STAT3 and its target, TFCP2L1, in self-renewal of naïve pluripotent stem cells in vivo and in vitro (Jennifer Nicolas 2022 bioRxiv). “Moreover, transfection of Tfcp2l1 into epiblast stem cells derived from postimplantation epiblasts (Brons et al., 2007; Tesar et al., 2007) could direct reprogramming from primed to naïve pluripotency, confirming participation of TFCP2L1 in the naïve pluripotency.” # network (Martello et al., 2013; Ye et al., 2013). # BM has WB in Wes # PubMed:25215486, 26906118. |  |  |  |  | List in Valamehr; Takashima, Theunissen |
| ***TFE3*** | Transcription Factor Binding to IGHM Enhancer 3 | A_23_P84952 | 1.11 | 0.88 | 1.40 | Takashima |
| ***UTF1*** | Undifferentiated Embryonic Cell Transcription Factor 1 | A_23_P33141 | 1.43 | 1.50 | 1.36 | Llist in Valamehr; mESC specific, Theunissen |
| ***XIST*** | X Inactive Specific Transcript | A_24_P500584 | 1.09 | 1.11 | 1.06 | P list in Valamehr |
| ***ZFP42*** | ZFP42 Zinc Finger Protein, known as Rex1, a definitive naïve marker in the mouse | A_23_P395582 | 0.83 | 0.87 | 0.79 | High in Takashima, Theunissen; Low in Gafni, Chan, & Ware PSCs |
| ***ZNF729*** | Human naïve transcriptomic marker, reviewed by Zhou J et al. Cell Reports, 2023. |  |  |  |  |  |
| *ZNF600* | Zinc Finger Protein 600 | A_23_P16006 | 1.22 | 1.01 | 1.48 | Epiblast specific |
| **Formative (between pre-implantation and the E6.5 epiblast in mouse) gene expression** (n = 5, * indicates *P* < 0.05) |  |  |  |  |  |  |
| ***DNMT3A*** | DNA Methyltransferase 3 Alpha **Transcriptionally regulated with naïve protocols, decrease in naïve** | A_23_P154500 | 0.98 | 0.97 | 0.98 | Smith review, Gafni |
| ***DNMT3B*** | DNA Methyltransferase 3 Beta **Transcriptionally regulated with naïve protocols, decrease in naive** | A_23_P28953 | 0.99 | 0.96 | 1.02 | Smith review; Decreased in Takashima, Gafni, & Neri |
| ***OTX2*** | Orthodenticle Homeobox 2 **Transcriptionally regulated with naive protocols** | A_23_P48663 | **0.52*** | 0.52 | 0.52 | Formative state, priming marker (Smith); Chan, Gafni, Theunissen, Valamehr, Ware |
| ***SALL2*** | Spalt Like Transcription Factor 2 | A_23_P48585 | **0.74*** | **0.78*** | 0.59 | Smith review |
| ***SOX3*** | SRY-Box Transcription Factor 3 | A_23_P85218 | 0.60 | 0.82 | 0.44 | Smith review |
| **Primed or metastable lineage specifier gene expression** (n = 37, * indicates *P* < 0.05) |  |  |  |  |  |  |
| *ACTC1* | Actin Alpha Cardiac Muscle 1 | A_23_P205894 | **0.09*** | **0.10*** | **0.08*** |  |
| *BMP2* | Bone Morphogenetic Protein 2 | A_23_P143331 | **0.28*** | 0.20 | 0.40 |  |
| *CD47* | CD47 Molecule | A_23_P6935 | 0.77 | 0.82 | 0.72 |  |
| *CDH11* | Cadherin 11 | A_23_P152305 | 1.33 | 1.54 | 1.15 | P list in Valamehr |
| ***CER1*** | Cerberus 1, DAN Family BMP Antagonist | A_23_P329798 | **0.27*** | 0.34 | **0.22*** | High in EpiSCs (Tesar & Brons); low in Chan & Takashima PSCs |
| *COL13A1* | Collagen Type XIII Alpha 1 Chain | A_23_P1331 | **0.28*** | 0.27 | **0.28*** | P list in Valamehr |
| *CXCR4* | C-X-C Motif Chemokine Receptor 4 | A_23_P102000 | **0.19*** | **0.15*** | 0.25 | P list in Valamehr |
| *CYP2B6* | Cytochrome P450 Family 2 Subfamily B Member 6 | A_24_P339514 | **0.32*** | 0.40 | 0.25 | P list in Valamehr |
| *DLL1* | Delta Like Canonical Notch Ligand 1 | A_23_P167920 | **2.86*** | 2.29 | 3.57 |  |
| ***DNMT1*** | DNA Methyltransferase 1 | A_24_P408083 | 0.90 | 0.80 | 1.01 | Zhao |
| ***EGR1*** | Early Growth Response 1 | A_23_P214080 | **0.01*** | **0.01*** | **0.01*** | P list in Valamehr |
| *EOMES* | Eomesodermin | A_24_P97374 | **0.18*** | 0.09 | 0.36 |  |
| *ERBB4* | Erb-B2 Receptor Tyrosine Kinase 4 | A_32_P183765 | **0.08*** | **0.07*** | **0.09*** | P list in Valamehr |
| *FLT1* | Fms Related Receptor Tyrosine Kinase 1 | A_24_P576191 | **0.22*** | 0.34 | 0.15 |  |
| *FOXA2* | Forkhead Box A2 | A_24_P365515 | **0.15*** | **0.07*** | 0.36 |  |
| *GAL* | Galanin And GMAP Prepropeptide | A_23_P374844 | **0.22*** | **0.18*** | 0.27 | P list in Valamehr |
| *GSC* | Goosecoid Homeobox | A_23_P76774 | **0.07*** | **0.06*** | **0.08*** | P list in Valamehr |
| *HEPH* | Hephaestin | A_24_P399980 | **0.74*** | 0.73 | 0.74 | P list in Valamehr |
| *HES1* | Hes Family BHLH Transcription Factor 1 | A_23_P17998 | **0.26*** | 0.31 | 0.22 | P list in Valamehr |
| *HHEX* | Hematopoietically Expressed Homeobox | A_23_P47034 | 0.70 | 0.50 | 0.97 | Pera & Rossant |
| ***LEFTY1*** | Left-Right Determination Factor 1 | A_23_P160336 | **0.04*** | 0.08 | 0.02 | P list in Valamehr Mallon, Chen |
| ***LEFTY2*** | Left-Right Determination Factor 2 | A_23_P137573 | **0.18*** | 0.27 | 0.13 | Mallon, Chen |
| *MEGF10* | Multiple EGF Like Domains 10 | A_24_P7192 | 1.74 | 2.15 | 1.41 | P list in Valamehr |
| ***MEIS1*** | Meis Homeobox 1 **Transcriptionally regulated with naive protocols** | A_24_P319736 | 1.24 | 1.55 | 1.00 | Low in Gafni PSCs |
| ***MEIS2*** | Meis Homeobox 2 **Transcriptionally regulated with naive protocols** | A_24_P82200 | 1.59 | 1.62 | 1.56 | Low in Gafni PSCs |
| *NIPBL* | NIPBL Cohesin Loading Factor | A_24_P180383 | 0.83 | 0.81 | 0.85 | P list in Valamehr |
| ***NODAL*** | **Transcriptionally regulated with naive protocols** # “NODAL Secures Pluripotency upon Embryonic Stem Cell Progression from the Ground State “Mulas, Kalkan, Smith. SCR. 2017” |  |  |  |  | Pera & Rossant |
| *NR2F2* | Nuclear Receptor Subfamily 2 Group F Member 2 | A_23_P88589 | 1.33 | 1.76 | 1.00 | P list in Valamehr |
| *PCDH10* | Protocadherin 10 | A_32_P168605 | 0.79 | 0.63 | 1.00 | P list in Valamehr |
| ***PITX2*** | Paired Like Homeodomain 2 | A_23_P167367 | **0.06*** | 0.08 | **0.05*** | P list in Valamehr |
| *RORA* | RAR Related Orphan Receptor A | A_23_P26124 | 0.58 | 0.70 | 0.48 | P list in Valamehr |
| *SOX1* | SRY-Box Transcription Factor 1 | A_24_P112803 | 1.37 | 1.37 | 1.37 | Pera & Rossant |
| *SOX11* | SRY-Box Transcription Factor 11 associated with neuron differentiation | A_24_P302584 | 1.30 | 1.40 | 1.21 | Low in Gafni PSCs |
| *TGFB2* | Transforming Growth Factor Beta 2 | A_24_P148261 | **0.69*** | 0.82 | 0.58 |  |
| *TOX* | Thymocyte Selection Associated High Mobility Group Box | A_24_P226755 | 1.21 | 1.33 | 1.10 | P list in Valamehr |
| *ZIC1* | Zic Family Member 1 | A_23_P367618 | 1.00 | 1.00 | 1.00 | Low in Gafni PSCs |
| *ZIC2* | Zic Family Member 2 | A_23_P36972 | 0.83 | 0.89 | 0.77 | Priming marker (Smith) |
| **Other X-lined or imprinted gene expression** (n = 5, * indicates *P* < 0.05) |  |  |  |  |  |  |
| ***DLK1*** | PEG9, Paternally Expressed 10, Known as DLK1, Delta Like Non-Canonical Notch Ligand 1 | A_24_P236251 | 3.41 | 2.56 | 4.54 | Low in naive & high in primed states in Gafni |
| *FMR1* | FMRP Translational Regulator 1, X-linked | A_24_P93967 | 1.30 | 1.80 | 0.93 |  |
| ***H19*** | H19, Imprinted Maternally Expressed | A_24_P52697 | 1.60 | 0.90 | 2.87 |  |
| ***MEG3*** | MEG3, Paternally Imprinted gene, Known as GTL2 **Transcriptionally regulated with naive protocols** | A_24_P272993 | 1.52 | 1.25 | 1.85 | High in naïve (Collier) |
| ***MEST*** | MEST, Mesoderm specific, imprinted, preferentially paternally expressed | A_23_P156970 | 0.92 | 0.92 | 0.93 |  |
|  |  |  |  |  |  |  |
|  | **The effects on SSPE** |  |  |  |  |  |
| ***1. control*** |  |  |  |  |  |  |
| ***2. SFRP1*** | **Secreted frizzled related protein 1** This gene encodes a member of the SFRP family that contains a cysteine-rich domain homologous to the putative Wnt-binding site of Frizzled proteins. Members of this family act as **soluble modulators** of Wnt signaling; **epigenetic silencing of SFRP** genes leads **to deregulated activation** of the Wnt-pathway which is associated with cancer. This gene may also be involved in determining the polarity of photoreceptor cells in the retina. [provided by RefSeq, Sep 2009]. |  |  |  |  |  |
| ***3. CER1*** | # This gene encodes a cytokine member of the cysteine knot superfamily, characterized by nine conserved **cysteines** and a cysteine knot region. The cerberus-related cytokines, together with Dan and DRM/Gremlin, represent a group of bone morphogenetic protein (**BMP) antagonists** that can bind directly to BMPs and inhibit their activity. [provided by RefSeq, Jul 2008] # by BT Dodsworth · 2020 · Cited by 14 — The primed marker gene **CER1** was upregulated within the first 5 days, whereas **OTX2** was only heavily upregulated upon acquisition of the primed ... ‎Cell Culture · ‎Results and Discussion · ‎Mirnas In Naïve To Primed... **# Mapping the route from naive pluripotency to lineage specification** Tüzer Kalkan1 and Austin Smith1,2 # https://www.nature.com › nature communications › articles by S Warrier · 2017 · Cited by 70 — 6b) **whereas more primed pluripotency-associated genes such as** DUSP8, CACNA1A, HGF, **CER1** ... (b) Genes related to naive pluripotency upregulated… # TGFβ signalling is required to maintain pluripotency of human naïve pluripotent stem cells Anna OsnatoStephanie BrownChristel KruegerSimon AndrewsAmanda J CollierShota NakanohMariana Quiroga LondoñoBrandon T WesleyDaniele MuraroPeter J Rugg-Gunn Is a corresponding author see all #,https://academic.oup.com › stmcls › article by MG Carter · 2016 · Cited by 39 — Systematic identification of culture conditions for induction and maintenance of naive human pluripotency ... **CER1 is a common target of WNT and NODAL** # Establishment of a primed pluripotent epiblast stem cell in FGF4-based conditions EpiSCs also expressed higher levels of epiblast markers, such as Cer1, Eomes, Fgf5, Sox17, and T, and further showed complete DNA methylation of Stella and Dppa5 promoters. |  |  |  |  |  |
| ***4. LEFTYA*** |  |  |  |  |  |  |
| ***5 DKK1*** | # **DKK1 dickkopf WNT signaling pathway inhibitor 1** [ Homo sapiens (human)] Gene ID: 22943, updated on 25-Dec-2023 # The **Dickkopf-1 (DKK1**) is a secreted inhibitor of beta-catenin-dependent Wnt signalling and was originally characterized as a tumour suppressor based on the prevailing view that Wnt signalling promotes cancer pathogenesis. **# Summary:** This gene encodes a member of the dickkopf family of proteins. Members of this family are secreted proteins characterized by two cysteine-rich domains that mediate protein-protein interactions. The encoded protein binds to the **LRP6 co-receptor** and **inhibits beta-catenin-dependent** **Wnt** signaling. This gene plays a role in embryonic development and may be important in bone formation in adults. Elevated expression of this gene has been observed in numerous human cancers and this protein may promote proliferation, invasion and growth in cancer cell lines. [provided by RefSeq, Sep 2017] |  |  |  |  |  |
| ***6 CER1+LEFTYA*** |  |  |  |  |  |  |
| ***7 CER1+DKK1*** |  |  |  |  |  |  |
| ***8 SFRP5*** | **# Secreted frizzled-related protein 5** (SFRP5) is a member of the SFRP family that contains a cysteine-rich domain homologous to the putative Wnt-binding site of Frizzled proteins. **SFRPs act as soluble modulators of Wnt signaling**. SFRP5 and SFRP1 may be involved in determining the polarity of photoreceptor cells in the retina. SFRP5 is highly expressed in the retinal pigment epithelium, and moderately expressed in the pancreas. [provided by RefSeq, Jul 2008] |  |  |  |  |  |
| ***9 BMP2+BMP4*** |  |  |  |  |  |  |
| ***10 BMP4*** | # BMP4 drives primed to naïve transition through PGC-like state.  Shengyong Yu et al 2022 # BMP4 resets mouse epiblast stem cells to naive pluripotency through ZBTB7A/B-mediated chromatin remodelling. NCB 2020. |  |  |  |  |  |
| ***11 BMP2*** | UniProtKB/Swiss-Prot Summary for BMP2 Gene **Molecular function for BMP2 Gene according to UniProtKB/Swiss-Prot** ***Function:*** Growth factor of the **TGF-beta** superfamily that plays essential roles in many developmental processes, including cardiogenesis, neurogenesis, and osteogenesis (PubMed:[18436533](https://pubmed.ncbi.nlm.nih.gov/18436533/), [31019025](https://pubmed.ncbi.nlm.nih.gov/31019025/), [24362451](https://pubmed.ncbi.nlm.nih.gov/24362451/)). Induces cartilage and bone formation (PubMed:[3201241](https://pubmed.ncbi.nlm.nih.gov/3201241/)). Initiates the canonical BMP signaling cascade by associating with type I receptor **BMPR1A** and type II receptor **BMPR2** (PubMed:[15064755](https://pubmed.ncbi.nlm.nih.gov/15064755/), [17295905](https://pubmed.ncbi.nlm.nih.gov/17295905/), [18436533](https://pubmed.ncbi.nlm.nih.gov/18436533/)). Once all three components are bound together in a complex at the cell surface, BMPR2 phosphorylates and activates BMPR1A (PubMed:[7791754](https://pubmed.ncbi.nlm.nih.gov/7791754/)). In turn, **BMPR1A** propagates signal by phosphorylating **SMAD1/5/8** that travel to the nucleus and act as activators and repressors of transcription of target genes. Also acts to promote expression of **HAMP**, via the interaction with its receptor BMPR1A/ALK3 (PubMed:[31800957](https://pubmed.ncbi.nlm.nih.gov/31800957/)). Can also signal through non-canonical pathways such as **ERK/MAP** kinase signaling cascade that regulates osteoblast differentiation (PubMed:[20851880](https://pubmed.ncbi.nlm.nih.gov/20851880/), [16771708](https://pubmed.ncbi.nlm.nih.gov/16771708/)). Stimulates also the differentiation of myoblasts into osteoblasts via the **EIF2AK3-EIF2A-ATF4** pathway by stimulating **EIF2A** phosphorylation which leads to increased expression of **ATF4** which plays a central role in osteoblast differentiation(PubMed:[24362451](https://pubmed.ncbi.nlm.nih.gov/24362451/)). [BMP2_HUMAN,P12643](https://www.uniprot.org/uniprot/P12643) |  |  |  |  |  |

**FOOTNOTES**

^a ^Based on the references as detailed in the reference list below. 

^**b**^ Transcriptionally regulated genes are indicated in bold gene symbols, with up-regrated gene in red and down-regulated gene in blue colors.

***** Indicates *P* values < 0.05 with two-tailed Student *t*-test, for fold changes of gene expression in microarray.

Additional abbreviations: EPS, epiblast specific gene signature; mESCs, naive mouse embryonic stem cells; N, naive-related genes; nPSCs, naïve pluripotent stem cells; PSCs, pluripotent stem cells; P, primed or primed genes or cells.

**REFERENCES**

Neri, F. et al. Genome-wide analysis identifies a functional association of Tet1 and Polycomb repressive complex 2 in mouse embryonic stem cells. Genome Biol 14, R91 (2013). https://doi.org:10.1186/gb-2013-14-8-r91

Brons, I. G. et al. Derivation of pluripotent epiblast stem cells from mammalian embryos. Nature 448, 191-195 (2007). https://doi.org:10.1038/nature05950

Chan, Y. S. et al. Induction of a human pluripotent state with distinct regulatory circuitry that resembles preimplantation epiblast. Cell Stem Cell 13, 663-675 (2013). https://doi.org:10.1016/j.stem.2013.11.015

Collier, A. J. et al. Comprehensive Cell Surface Protein Profiling Identifies Specific Markers of Human Naive and Primed Pluripotent States. Cell Stem Cell 20, 874-890 e877 (2017). https://doi.org:10.1016/j.stem.2017.02.014

Gafni, O. et al. Derivation of novel human ground state naive pluripotent stem cells. Nature 504, 282-286 (2013). https://doi.org:10.1038/nature12745

Liao, J. et al. Targeted disruption of DNMT1, DNMT3A and DNMT3B in human embryonic stem cells. Nat Genet 47, 469-478 (2015). https://doi.org:10.1038/ng.3258

Chen, K. G. et al. Non-colony type monolayer culture of human embryonic stem cells. Stem Cell Res 9, 237-248 (2012). https://doi.org:10.1016/j.scr.2012.06.003

Mallon, B. S. et al. StemCellDB: the human pluripotent stem cell database at the National Institutes of Health. Stem Cell Res 10, 57-66 (2013). https://doi.org:10.1016/j.scr.2012.09.002

Pera, M. F. & Rossant, J. The exploration of pluripotency space: Charting cell state transitions in peri-implantation development. Cell Stem Cell 28, 1896-1906 (2021). https://doi.org:10.1016/j.stem.2021.10.001

Smith, A. Formative pluripotency: the executive phase in a developmental continuum. Development 144, 365-373 (2017). https://doi.org:10.1242/dev.142679

Takashima, Y. et al. Resetting transcription factor control circuitry toward ground-state pluripotency in human. Cell 158, 1254-1269 (2014). https://doi.org:10.1016/j.cell.2014.08.029

Tesar, P. J. et al. New cell lines from mouse epiblast share defining features with human embryonic stem cells. Nature 448, 196-199 (2007). https://doi.org:10.1038/nature05972

Theunissen, T. W. et al. Molecular Criteria for Defining the Naive Human Pluripotent State. Cell Stem Cell 19, 502-515 (2016). https://doi.org:10.1016/j.stem.2016.06.011

Theunissen, T. W. et al. Systematic identification of culture conditions for induction and maintenance of naive human pluripotency. Cell Stem Cell 15, 471-487 (2014). https://doi.org:10.1016/j.stem.2014.07.002

Valamehr, B. et al. Platform for induction and maintenance of transgene-free hiPSCs resembling ground state pluripotent stem cells. Stem Cell Reports 2, 366-381 (2014). https://doi.org:10.1016/j.stemcr.2014.01.014

Weinberger, L., Ayyash, M., Novershtern, N. & Hanna, J. H. Dynamic stem cell states: naive to primed pluripotency in rodents and humans. Nat Rev Mol Cell Biol 17, 155-169 (2016). https://doi.org:10.1038/nrm.2015.28
